# The Evolution of Gambling-Related Harm Measurement: Lessons from the Last Decade

**DOI:** 10.3390/ijerph18094395

**Published:** 2021-04-21

**Authors:** Matthew Browne, Vijay Rawat, Catherine Tulloch, Cailem Murray-Boyle, Matthew Rockloff

**Affiliations:** School of Health, Medical and Applied Sciences, CQUniversity, Bundaberg 4670, Australia; v.rawat@cqu.edu.au (V.R.); c.tulloch@cqu.edu.au (C.T.); c.murrayboyle@cqu.edu.au (C.M.-B.); m.rockloff@cqu.edu.au (M.R.)

**Keywords:** gambling, measurement, public health, gambling harm, problem gambling

## Abstract

Jurisdictions around the world have a self-declared mandate to reduce gambling-related harm. However, historically, this concept has suffered from poor conceptualisation and operationalisation. However, recent years have seen swift advances in measuring gambling harm, based on the principle of it being a quantifiable decrement to the health and wellbeing of the gambler and those connected to them. This review takes stock of the background and recent developments in harm assessment and summarises recent research that has validated and applied the Short Gambling Harms Screen and related instruments. We recommend that future work builds upon the considerable psychometric evidence accumulated for the feasibility of direct elicitation of harmful consequences. We also advocate for grounding harms measures with respect to scalar changes to public health utility metrics. Such an approach will avoid misleading pseudo-clinical categorisations, provide accurate population-level summaries of where the burden of harm is carried, and serve to integrate gambling research with the broader field of public health.

## 1. Introduction

Although gambling-related harm (GRH) has been a topic of interest in the field for at least 15 years, there has been a recent increase in research specifically addressing this issue. Early work tended to rely on repurposed gambling problems measures and adopted a categorical classification of harm that is not much distinct from the concept of gambling problems as a mental health issue. Contemporary research has increasingly relied on dedicated measures, a more precise delineation of the distinction between indicators of behavioural addiction vs. harm, and focussed on the health-related impacts stemming from excessive consumption. It is possible to experience harm from gambling without being behaviourally addicted, and more rarely, it is at least possible to develop a behavioural addiction before suffering significant gambling losses. These developments have gone hand-in-hand with greater adoption of a public health framework to address questions such as the ‘prevention paradox’ (i.e., the finding of more population-level harm amongst the large group of gamblers with few individual-level gambling problems), the link between GRH and health and wellbeing (i.e., the association between gambling harm and decrements to quality of life), and the treatment of negative gambling impacts on a continuum from mild to severe (as opposed to categories of “harmed” and “not harmed”). At the same time, there have been significant disagreements and debate regarding both technical and conceptual aspects of this implementation of a public health approach to gambling, since it breaks with a long-standing tradition of research into problem gambling as a mental health disorder.

It is timely to review the state of play of developments in this area. Browne et al. [1] recently described a technical framework for evaluating GRH using propensity score matching as a means for addressing issues of comorbidities and risk factors, with the aim of linking GRH to established public health metrics such as the SF-6D [2]. A theoretical and conceptual overview of the distribution of GRH in the population is given by Browne [3]. However, neither of these overview articles attempted to provide a narrative description of the evolution of research into the measurement and evaluation of GRH. Raybould et al. [4] conducted a systematic review focused on health inequalities in the experience of GRH by age, gender, and other demographic factors. The review included English-language empirical studies employing dedicated measures of harms to the gambler but excluded studies on harms occurring to affected others, nor did it include harm assessed via legacy measures based on gambling problems. Instead, it focussed on demographic differences and the prevention paradox. It did not address the methodological or conceptual issues in the literature, such as distinguishing between gambling problems, gambling harm, and indicators of behavioural addiction, and how each should be measured. 

In contrast, frameworks and review papers on GRH (e.g., [5,6,7]) have provided a strong conceptual framework for understanding what constitutes gambling harm. Still, they have not focussed on the methodological issues of measurement. Accordingly, the present review will take stock of recent developments in the measurement of GRH as experienced by gamblers, as well as the use of the concept. We discuss controversies associated with this approach and consider applications to key public health questions in gambling research.

## 2. Measuring Harm from Gambling

The following subsections describe the practice of GRH assessment over the last 15 years. We shall argue that the early measurement of GRH was limited. It repurposed problems-based measures and therefore conflated the consequences of excessive gambling (i.e., harms) with the behavioural risk factors for excessive gambling (pre-occupation, lack of control, and so on). Perhaps most importantly, framing the measurement of GRH to the criteria for a clinical diagnosis leads to the seductive mistake of all-or-none categorization. This contrasts with the far more realistic view that harms and benefits occur as a matter of degree. Finally, early approaches tended to ignore the definition of GRH as being an impact on one’s health and wellbeing attributable to gambling. As a result, until recently, no validation of GRH was done to this key benchmark. However, as the narrative review below illustrates, there has been a steady trend towards addressing these deficits.

### 2.1. Repurposing Problems-Based Measures

A substantial proportion of GRH research has employed items from the Problem Gambling Severity Index (PGSI), a subset of the Canadian Problem Gambling Index (CPGI), to measure harm. The CPGI includes a number of negative consequences that can be understood as harms, e.g., health problems including stress or anxiety, financial problems, borrowing money, or selling possessions to gamble, interpersonal problems and feeling guilty about gambling. These items were used effectively by Canadian studies focussed on estimating risk curves associated with increasing gambling consumption [8,9], following the dose–response model that has been applied successfully to the epidemiology of alcohol-related harm. In deriving low-risk gambling limits, Currie et al. [10] also relied on harm classifications based on the PGSI. Likewise, in estimating the impact of gambling losses, Markham et al. [11] used the South Oaks Gambling Screen (SOGS), PGSI, and the NORC DSM-IV Screen for Gambling Problems (NODS) [12], treating higher scores as indications of higher levels of harm. In a population-based survey, Canale et al. [13] employed 10 harm items adapted from the DSM-IV Pathological Gambling criteria. These were grouped as dependence harms (e.g., impaired control), possible dependence harms (e.g., chasing losses), and social harms (e.g., relationship or legal problems). Other studies have used a measure such as the PGSI essentially as-is, expediently treating it as an index of harm [14].

Thus, the studies mentioned above have used measures of problem gambling as proxies for harm. Although some of the items in such scales are clearly harmful consequences of gambling, other items may arguably be indicators of behavioural addiction or even simply incidentally related to gambling harm (e.g., returning another day to win back money lost at gambling). The most pernicious effect of this practice is that the conceptualisation of gambling harm has ‘inherited’ the clinical categorisation model of gambling problems, obscuring the underlying reality that most people highly involved in gambling experience some benefits as well as some degree of harm from their gambling.

### 2.2. Bespoke/Custom Approaches

Rather than repurposing existing problems-based measures, Raisamo et al. [15] took a more systematic approach. A pool of eight harms covering multiple domains were selected based on a literature review and expert consultations. For each harm, respondents also nominated the frequency of harms (e.g., daily/almost daily). In this 2013 study, and a 2020 follow-up [16], a combined variable was created for analysis with three categories: no harms, one harm, and more than one harm. The researchers found that experiencing harms was positively associated with gambling frequency [15], and the most common harm reported was “feeling guilty or ashamed” [16].

In a different study of gambling impact on Indigenous Australians, Hing et al. [17] used 11 binary (yes/no) items that covered consequences such as depression, job loss, criminal activity, and bankruptcy. All items were strong discriminators of differing levels of gambling problems as indexed by the PGSI. For example, ‘arguments within your household’ (over gambling) had a prevalence rate of 0.9% among non-problem gamblers (NP), rising to 44.7% among problem gamblers (PG). In addition, the more severe consequence of separation or divorce rose from 0.3% (NP) to 21.5% (PG). These examples help to illustrate a seemingly paradoxical property of gambling harms: that ‘milder’ consequences, because they are more prevalent, are often a more reliable index of severity than severe ones. That is, many mild harmful consequences are almost ubiquitous amongst people with severe gambling problems, whereas the more severe negative consequences are more idiosyncratic.

Salonen et al. [18] analysed data from two existing Finnish gambling surveys from 2011 (*N* = 4484) and 2015 (*N* = 5415). A total of 16 harm items were derived from the SOGS and the PGSI, with binary scoring indicating the presence (or not) of that harm. Although men reported more harms than women, the most commonly reported across both genders were gambling more than intended, chasing losses, and feelings of guilt. However, as discussed in Browne and Rockloff [19], the first two probes arguably describe behavioural addiction rather than a direct impact on health and wellbeing.

Using the large (*N* = 3325) Quinte Longitudinal Survey (QLS) data, Cowlishaw et al. [20] conducted a sophisticated item response theoretic (IRT) analysis of a similar combined set of items from the PGSI, the NODS, and the Problem and Pathological Gambling Measure (PPGM) [21]. They confirmed that both problematic gambling behaviours and specific harmful consequences lie on a single continuum of severity. However, they also comment that most of their available probes tended to lie within a relatively narrow band on the severity continuum. Interestingly, harms such as significant health, work or school problems, or family neglect and criminal activity, rather than indicators of addiction, reflected the most severe end of this spectrum. Most recently, Stevens et al. [22] employed a custom list of 16 negative consequences attributable to gambling, providing good coverage of a range of financial, relationship, and psychological/behavioural issues, and binary (yes/no) scored. One-quarter of at-risk gamblers reported some kind of harm, with the most commonly reported harms being financial and psychological impacts.

Stevens et al. [22] found that mild–moderate harms, in aggregate, were substantially more numerous in larger non-clinical gambling populations. This underlines the idea that assessing differing degrees of GRH is necessary to obtain a realistic estimate of the total population impact of gambling. On one hand, it is safe to assume that most harmed gamblers are not experiencing harm close to the degree experienced by problem gamblers. On the other hand, discounting the impacts to this far larger group ignores an important part of the spectrum that contributes to total societal impact.

### 2.3. Health–Economic Assessment

Health–economic approaches support the idea that GRH should be approached by considering all impacts across the population. Unlike the psychometric self-report paradigm favoured by psychologists, these methods include numerous estimates of costs at the population level, including items such as costs of bankruptcies, divorce, or lost productivity, as well as less tangible indicators. Just as when measuring the impacts of social issues such as domestic violence or alcohol misuse, there is no assumption that impacts are restricted to ‘alcoholics’ or people whose actions pass some threshold for violence intensity. Although an economic framework has a natural preference for hard indicators easily quantified in economic terms, it is recognised that the most important impacts of gambling are likely in terms of health and wellbeing, which are experienced individually and sometimes subjectively [23,24].

A comprehensive review of gambling health–economic studies is beyond the scope of this article, but the framework of Wardle et al. [25] provides an illustrative and representative example. They organise metrics relating to GRH in a social costing framework relating to limitation of resources (work and employment, money and debt, crime), relationships (familial and community participation), and health (physical, psychological wellbeing/happiness, and mental health). In general, the lists of specific harmful consequences enumerated within various costing frameworks overlap to a large degree [7,24,25,26], suggesting there is quite strong agreement regarding the possible adverse outcomes from gambling problems. The Australian Productivity Commission [27] costing study has arguably formed the model for most subsequent efforts. It employed 22 items describing gambling harms across six domains, measured in a binary format (yes/no), with wording specifying a causal attribution between gambling and harm. Finally, McDaid and Patel [28] also outline a measurement framework with social costing in mind, recommending, among other things, the increased use of standardised health and wellbeing measures such as the SF-12 or SF-6D [29] in addition to gambling-specific outcomes.

There is an important and mutually beneficial relationship between health–economic costings and population-representative self-reports of GRH. The conceptual framework of health–economic costings supports the premise that self-report measures should be sensitive to degree and grounded in recognised common metrics, such as the SF-6D [1] or disability weights [30]. Similarly, it is difficult or impossible for health–economic studies to integrate self-report measures of intangible costs, e.g., psychological wellness or relationship health, that are not linked to a meaningful health utility metric. In other words, knowing that 2.7% of the population are estimated to be ‘moderate-risk gamblers’ tells the economic modeller nothing about the degree of suffering those individuals are experiencing, and it provides no clear guidance on how to incorporate this information into estimates of intangible health and wellbeing impact.

### 2.4. Measures Targeted at GRH

The Victorian Gambling Screen (VGS) [31] was perhaps the earliest attempt to include a dedicated measure of the possible harmful consequences of gambling. It included 16 items intended to capture harm-to-self (HS). The HS subscale has been reported to perform well in relation to the SOGS in identifying problem severity, and having good internal consistency in a clinical population [32]. Unfortunately, the HS subscale includes a number of items, such as concealing gambling, urges, and returning to win back losses, which are better considered as indicators of behavioural dependence rather than harms. Furthermore, the item content in some cases is somewhat vague and therefore vulnerable to interpretive biases, such as feeling as if one was “on a slippery slope”. This is reflected in low loadings (around 0.50) on the HS factor [33]. Overall, the VGS performed poorly compared to the PGSI and the SOGS [34]. Although the VGS was introduced almost 20 years ago, it appears not to have had widespread adoption in gambling research.

The Problem and Pathological Gambling Measure (PPGM) is a 17 item measure that groups items into three categories: ‘problems’ (harms), ‘impaired control’, and ‘other issues’ [21]. The problems items are a mixture of Likert and binary scored items, capturing concepts such as financial concerns, mental stress, and conflict with family and friends [35]. Strong psychometric performance has been reported for the PPGM [21,36,37]. Christensen et al. [38] conducted a factor analysis of a large combined dataset of items from the NODS, PGSI, and PPGM. They found four components: (1) general gambling problems, (2) financial impacts, (3) relationship and health impacts, and (4) symptoms of behavioural addiction. The statistical separation of behavioural dependence and adverse effects is consistent with what was found by Browne and Rockloff [19]. It also agrees with domain-level analyses of harms done by Browne et al. [39], which suggested that financial problems comprised the earliest and most reliable outcome of excessive gambling consumption. Relationship and health problems subsequently follow in some (but not all) cases, depending on the severity of the gambling problem and the personal situation of the gambler.

The Harm Questionnaire (HQ) [40,41] represents a nuanced approach to assessing GRH. Items were derived via a systematic process, sourcing eight items of harm organised in seven domains from the literature. Each harm is probed via two items, each with a 5-point Likert response. The first question involves reporting the degree to which the issue (e.g., drug use) has been a problem in one’s life. The second question asks about the degree to which the problem was related to one’s gambling. This contrasts with other self-report measures reviewed here, in which attribution to gambling is all-or-none, and the adverse consequence is often reported as present or absent. The tool has since been employed to make community and clinical comparisons [42]. The HQ reflects one approach to an important unresolved problem in GRH assessment, which is “...how to quantify empirical units of gambling-related harm” [41] (p. 378) and move away from the present reliance on problem gambling status as a proxy for harm. Consistent with other literature, they found that psychological and financial harms are the most significant widespread impacts. In addition, consistent with other research [7,43] was the finding that the most severe impacts at the community level were general impacts on quality of life, rather than ‘crisis’ harms such as suicide or bankruptcy.

### 2.5. The Short Gambling Harms Screen

The 10-item Short Gambling Harms Screen (SGHS) [44] was developed from the much longer 72 item ‘harms checklist’ employed in prior studies to assess the impact of gambling on health and wellbeing [45,46]. Although the original checklist covered six broad domains of impact (financial, health, relationships, psychological, social deviance, work/study), the binary (yes/no) items were selected based purely on statistical criteria to maximise sensitivity and specificity without regard for item content. The SGHS has been criticised for not covering all domains of harm, emphasising milder items (e.g., reduction in savings), and for its binary present/absent scoring approach [47,48,49]. Nevertheless, it has been shown to possess good to excellent psychometric properties and has had the widest uptake among gambling harm measures, particularly in Australia where it was developed. Table 1 summarises research that has evaluated or applied the SGHS since its development.

As shown in Table 1, the initial psychometric validation of the SGHS [44] has been followed by several more papers focusing on specific criticisms of the instrument. McLauchlan et al. [50] considered both binary and Likert response formats for the SGHS and the PGSI, and found approximately equivalent performance for each format. McLauchlan and colleagues also calculated correlations between the SGHS and psychological distress and personal wellbeing (PWI) across the test and re-retest waves. They reported high (0.6 to 0.7) correlations between the SGHS and both outcomes, with no significant difference between the two response formats. Murray-Boyle et al. [51] focused on the milder probes within the SGHS (e.g., ’reduction in savings’), finding them to be reliable and valid indicators of ‘unimpeachably’ severe harms (e.g., social isolation, feelings of worthlessness, and being absent from work or study). Using different population-representative data from Victoria, Murray-Boyle et al. [52] considered the relationship of SGHS scores with self-reported wellbeing (using the Australian Unity Wellbeing Index, AUWI) and (Kessler) distress. Gamblers with SGHS scores of zero were identical to non-gamblers. However, SGHS scores of 1+ demonstrated statistically significant decrements, with these becoming incrementally more severe as SGHS scores increased. Table 1 also enumerates the 17 documented applications of the SGHS available at the time of writing.

### 2.6. Reflections on the Evolution of Self-Reported GRH

A theoretical limitation of early ad hoc measurement of harm has been the combination or conflation of indicators of behavioural addiction, with the consequences of excessive time or money expenditure. If harms are conceptualised as involving a direct decrement to an individual’s health and wellbeing, then the inclusion of behavioural dependence items presents an issue in terms of content validity. However, because behavioural dependence is so strongly statistically coupled with excessive consumption and harm, this may not be a significant practical problem in many applications. As has been noted by Currie et al. [8,9], the selection of items and/or classification as ‘being harmed’ creates uncertainty as to what threshold should constitute genuine harm. This issue is exacerbated when a small set of harms is sourced from legacy measures such as the PGSI, and it is repurposed to classify gamblers into ‘harmed or not’ categories. The work of Cowlishaw et al. [20] goes a long way to clarifying this issue, in utilising a large set of candidate indicators, and recognising that specific adverse consequences lie on a continuum. From this point of view, specific observed symptoms reflect differing degrees of underlying impact rather than category membership. Cowlishaw et al. [20] further suggest that items targeting the lower end of the continuum are under-represented in existing measures, and they recommend continued development of a pool of lower-severity cognitive–affective and behavioural items.

The PPGM, the HQ, and the SGHS can be thought of as the ‘next generation’ of measures that aim to specifically assess GRH. The HQ innovates with an interesting two-step approach that attempts to address the degree to which a given harm is attributable to gambling. This contrasts with the more typical self-attribution approach taken by the PPGM and the SGHS, which instruct respondents to respond to items only with respect to gambling. Although the HQ is currently lacking psychometric evidence for efficacy, this approach warrants further attention. Both the PPGM and SGHS have had good uptake and published psychometric validation. Conceptually, they differ primarily in terms of whether they are presented as providing a rule for categorical determination (PPGM) versus eschewing categories in favour of a dimensional measure (SGHS).

A deficiency of all available measures is the lack of a confirmed metric to capture the ‘units’ of GRH. In our view, the only meaningful quantum of measurement is in terms of expected decrements to health utility, as captured by either self-report instruments such as the SF-6D or via direct elicitation as disability weights. Health utility is the public health concept whereby ideal health and wellbeing is defined as unity (1), with the other pole of zero (0) describing an intolerable life that is not worth living. Although work has been done to relate both the PGSI and the SGHS to health utility decrements [30,46,53], this needs to be repeated more explicitly in the context of psychometric scale development.

## 3. Effects of GRH on Health and Wellbeing

There is one overriding reason why we should care about GRH, and that is because it leads to measurable decreases in people’s health and wellbeing. However, early employment of the construct made no attempt to validate this purported impact using external measures. For example, when attempting to define a gambling consumption threshold for harm, Currie et al. [9] assumed that ‘being harmed’ was a threshold that needed to be met by experiencing an arbitrary mix of consequences and behaviours. A similar frame is still evident in more recent research, in which multiple thresholds for ‘being harmed’ are evaluated, without a clear conclusion as to which threshold is preferred [49]. Again, this appears to reflect a conceptual cul-de-sac that assumes the need for categorisation that is inherited from clinically inspired problem gambling instruments. Whereas diagnostic instruments are best ground-truthed via clinical interviews, GRH is better validated via recognised measures of health utility.

Although direct psychometric validation is still scant, there is much indirect evidence that GRH is a coherent construct that affects health and wellbeing. In a study of 1259 indigenous Australian gamblers, Hing et al. [17] found that depression and household arguments were the most prevalent consequences among problem gamblers. On the other hand, when the SGHS was administered to a large sample of 5076 online wagerers, the most common reported harms were ‘reduction of my available spending money’ (23.5%), ‘reduction of my savings’ (21.5%), and ‘had regrets that made me feel sorry about my gambling’ (18%) [59]. Salonen et al. [69] employed the 72-item harm checklist [7] (from which the SGHS was derived) in a large-scale Finnish survey that included both population and clinical samples. The most common harms reported in the population sample were financial and emotional/psychological harms. These were also the most prevalent forms of GRH reported in the clinical sample, but this group also reported a relatively high number of harms associated with health and relationships.

In our view, health utility provides an essential grounding for evaluating the diverse and variable impacts from gambling. This echoes exhortations by others [4] to follow a harm minimisation paradigm, to “...consider aggregate harm to individuals, rather than the estimated prevalence of problem gamblers”. Rockloff et al. [70] used the time trade-off (TTO) elicitation approach to assess benefits and negative impacts on both gamblers and concerned significant others (CSOs). They found that gambling likely yields a negative net consumer surplus for Tasmanians. This followed earlier work [30,46] that estimated health utility for each of the PGSI categories. Rockloff et al. [63] applied similar utility weights to the SGHS in a Victorian population study (*N* = 10,638) to calculate aggregate health impacts of GRH. In Victoria, Australia, they calculated a decrement of 0.44 for problem gamblers, with smaller decrements for moderate (0.29) or low-risk gamblers (0.13). The Tasmanian population prevalence study, noted above, also found that health utility had a negative relationship to the SGHS when using a sequential discrete-choice TTO protocol [53]. This is consistent with the approximately linear relationship between the SGHS and subjective wellbeing reported by Browne et al. [44] and similar to effects noted for the PGSI, with subjective wellbeing decreasing linearly with increasing risk status [71]. Finally, Murray-Boyle et al. [52] used Victorian population prevalence data to demonstrate two important findings. First, Murray-Boyle et al. found that unharmed gamblers (SGHS = 0) showed statistically indistinguishable levels of (Kessler) distress and subjective wellbeing to non-gamblers. Second, results revealed that both outcomes deteriorated significantly and progressively with increasing GRH (SGHS 1+).

Ground-truthing measures of GRH to subjective wellbeing, psychological distress, or health utility is an important and ongoing program of research. However, available current evidence already indicates that instruments such as the SGHS are not only diagnostic of key outcomes but they are also able to differentiate differing degrees of GRH.

## 4. The Prevention Paradox

Quantifying harm in a population leads naturally to questions such as the prevention paradox (PP). The PP refers to a situation in which the majority of negative outcomes are attributable to a more populous group that do not exhibit a risk factor, as compared to a smaller group who do. To illustrate, the majority of alcohol-related problems (of varying severity) among adolescents were found to be accounted for by the bottom 90% of drinkers by alcohol intake [72]. The so-called ‘paradox’ in terms of population–aggregate impact arises simply because the increased risk at the individual level is more than counterbalanced by the lower prevalence of the risk factor.

While the PP has been a long-standing observation in other areas of public health, it was seldom considered in gambling until raised by Delfabbro and King [47], who cautioned against its application without considering “... some meaningful threshold for these behaviors and that they are seen to reduce people’s quality of life or compromise their psychological, physical, or social wellbeing” (p. 166). This hints at a fundamental issue in traditional PP reasoning; to calculate a relative proportion of incidents in the low and high-risk categories, the PP is only meaningful with a discrete outcome of interest, e.g., the occurrence of alcohol-related violence among problematic and non-problematic drinkers. The concern of Delfabbro and King [47] and others is that by setting a low-enough threshold for harm, the PP can always be confirmed, and the apparent societal impact of an activity such as gambling thereby exaggerated.

The issue may be partially resolved by considering the PP groupwise with respect to a broad range of outcomes across a spectrum of severity. This was undertaken by Browne and Rockloff [43], who evaluated the PP with all 72 harms in the harms checklist [7]. Using the PGSI to assess risk, they found that the majority of harms in the 72-item list were attributable to low- and moderate-risk gamblers rather than problem gamblers. These included relatively serious harms such as needing temporary accommodation, emergency welfare assistance, experiencing separation or end of a relationship, loss of a job, needing to sell personal items, and experiencing domestic violence from gambling. This analysis was repeated using a Finnish population survey [73] and using the PPGM control dimension to assess risk. This study found that most financial, emotional, and work/study impacts occurred to those with lower levels of control issues. However, most health, relationship, or social deviance harms tended to be attributable to those with more severe control issues. Another population study in New South Wales, Australia included 21 harm items from the moderate–severe end of the spectrum [74]. Aggregate calculations from these data indicated that approximately half of these harms were attributable to problem gamblers. Using NP gamblers as a baseline, Blackman et al. [71] found that discrepancies in Personal Wellbeing Index (PWI) scores implied that almost half of gambling harm (46.2%) was attributable to low-risk gamblers, 38.5% was attributable to moderate-risk gamblers, and 15.3% was attributable to problem gamblers. These attributable proportions were strikingly similar to estimates calculated using health utility disability weights using the TTO and VAS elicitation methods in Victorian and New Zealand studies [39,75]. In Tasmania, Australia, disability weights were empirically linked to the SGHS, and when aggregated, they found similar results, with 17.8% of utility decrements attributable to problem gamblers [53]. However, Delfabbro et al. [76] analysed data from an online panel of 554 gamblers, considering several alternative scoring strategies for classifying people as harmed or not. They found that determination of the proportion of harm—using thresholds for being harmed or not—that were attributable to the various risk categories depended greatly on where this threshold was set. However, the tendency to apply a fixed categorisation of “harmed” vs. “not harmed” may not be necessary or desirable. When harm is measured along a continuum and related to outcomes such as disability weights, as noted above, population decrements in these outcomes are invariably highly concentrated in the low-risk population of people with less-severe gambling problems.

Thus, in our view, the discourse around the PP in gambling is really a surrogate for the more important question of whether the impacts to health and wellbeing, i.e., harm, are concentrated in a few people with a severe pathology or more broadly distributed in the more typical gambling population. Any number of answers to the PP can be generated if one is selective as to which outcomes count as genuine harms or if one creates custom thresholds for categorising people as harmed or not. In sum, arbitrary outcomes and thresholds are more likely to mislead than not. PP logic is best suited to situations when there is a clear unitary outcome of interest that occurs or does not (e.g., laying of criminal charges) and clear categorisation of whether people belong in the risk category (e.g., diagnosed with diabetes or not). While these issues can be partially addressed by being comprehensive in the scope of outcomes measured, and considering multiple thresholds of risk (e.g., as done by Browne and colleagues [43,73]), such analyses do not directly address the issue of quantifying impact and mapping its distribution in the population.

## 5. Conclusions

Both definitions of problem gambling and public health epidemiological frameworks conceptualise impacts from gambling or harm as a scalar decrement to health and wellbeing-not a categorisation as ‘harmed or not’. Indeed, there is a consensus in the field that gambling consumption, behavioural dependence, and harm should all be thought of as continuous quantities [3]. Given this, it is surprising how little research in the discipline has attempted to assess this impact on a continuum, using recognised quality-of-life measures. These studies are distinct from evaluations of the PP, because they treat impact as a scalar decrement to wellbeing or health utility rather than calculating proportions of individuals as ‘suffering a harm or not’.

The next key goal in assessing GRH is to fully integrate established measures of gambling problems with the integrated public health Burden of Disease framework. Any self-report measure of GRH yields a numeric score, which must then be grounded to some meaningful assessment of what that score implies. This entails linking to a common metric of health utility (or DW), as discussed in detail by Browne et al. [1]. Within a public health Burden of Disease assessment system, clinically relevant categories (e.g., those derived from the PGSI) are related to typical DWs. Ideally, such assessments are integrated with other co-occurring conditions, so that the unique impact of gambling is factored out. Then, epidemiological population metrics calculated from these provide the cornerstone for rational public policy and intervention decisions. Although the ‘Burden of Harm’ studies [39,75] represent an important step in this project, the integration component that accounts for comorbidities (such as alcohol abuse) remains to be accomplished.

A second and related goal is to ground-truth scores from candidate GRH instruments to DWs. In our view, this is the only way to avoid circular arguments or intuitive and entirely subjective judgements regarding ‘how bad’ a given degree of measured GRH actually is. In terms of psychometric evaluation of alternative instruments, it seems clear—almost by definition—that the impact on health and wellbeing is the core benchmark of interest for GRH. Self-report measures of health and wellbeing, such as the SF-6D [2] or the EQ-5D [77], are the obvious first choice, perhaps supplemented by more general measures of wellbeing already in use, such as the PWI. As detailed in Browne et al. [1], this benchmarking would ideally be done via a matched sampling and weighting, so as to isolate the effect of GRH from comorbidities and correlates.

Based on the quality of psychometric validation and the number of applications, the SGHS is the clear front-runner candidate among current instruments for assessing GRH. Although the available psychometric and validation evidence is strong, it currently lacks formal evaluation that includes both a propensity model (i.e., comparing those reporting harm to an equivalent sample of those without harm) and a causal model (i.e., controlling for comorbid conditions). Furthermore, the SGHS was designed to be a brief unidimensional screen and to maximise sensitivity. The literature acknowledges six domains of harm, and a more comprehensive measure may provide advantages in some contexts. Finally, the elicitation of how much each symptom was caused by gambling (as opposed to other causes, as done by the HQ) may present a useful refinement.

In conclusion, we make the following recommendations. First, that further development of GRH measurement leverages psychometric evidence already established for the SGHS. The SGHS has been shown to possess excellent internal psychometric properties, be a good surrogate for the comprehensive 72-item screen, and discriminate differing degrees of GRH, using external benchmarks such as the PWI or the Kessler Distress Scale.

Our second recommendation is that formal health utility weights be established, both on a dimensional continuum (e.g., for SGHS or other GRH measures) and for clinical or pseudo-clinical categories (e.g., for PGSI scores). This should be done via a propensity score matching design, using established public health metrics as the key benchmark. The former will allow for accurate assessment of the distribution of differing degrees of GRH among subpopulations. The latter will facilitate the integration of GRH assessment within frameworks such as the Global Burden of Disease. With these two steps taken, GRH assessment can move beyond circular or subjective arguments around what should constitute harm and provide a firm foundation for future research and policy.

## Figures and Tables

**Table 1:** Overview of studies employing the SGHS.

Reference	Sample Size; Locality	Key Findings
Browne et al. [44] ^a^	1524; Australia	SGHS scores correlated with the full 72-item checklist at *r* = 0.94. Increasing SGHS scores predicted subjective wellbeing (*r* = −0.29) better than PGSI or addiction measures. Scale shown to be unidimensional and to have measurement invariance with respect to age and gender.
McLauchlan et al. [50] ^a^	532; US	The psychometric performance of the SGHS remained equivalent when changing the scoring format from binary to Likert scale. No significant differences were found among correlations between the binary and Likert versions of the SGHS and measures of psychological distress, impulsivity, and wellbeing.
Murray-Boyle et al. [51] ^a^	5551; Australia/New Zealand	The SGHS was strongly correlated with a range of measures, including the PGSI (*r* = 0.68), a latent gambling harm variable (*r* = 0.87), and a gambling harm scale only including unambiguously harmful consequences (*r* = 0.73). The findings lend support to the unidimensionality and reliability of the SGHS, particularly concerning the SGHS items capturing legitimate harmful consequences.
Murray-Boyle et al. [52] ^a^	1742; Victoria, Australia	When examining five SGHS items criticised as being non-genuine harms, this study found that endorsing any of the five items predicted lower wellbeing and higher psychological distress. Each item individually predicted declines in health-related quality of life, and endorsement of additional harm items was associated with cumulative declines.
Acil Allen Consulting et al. [53] ^b^	5000; Tasmania, Australia	Using the SGHS, 5531 years of life were lost due to gambling per annum, and this figure was similar when using the PGSI (5083 years of life lost). The mean number of harms increased along with PGSI categories: non-problem gamblers had a mean SGHS score of 0.057, 0.59 for low-risk gamblers, 2.164 for moderate-risk, and 5.565 for PGs.
Browne et al. [54] ^b^	1174; Canada	Similar proportions of respondents scored 1+ on the PGSI (48.6%) compared to non-zero responses on the SGHS (41.9%). The key proximal and distal risk factors for gambling harm were trait impulsivity, early childhood gambling exposure, gambling fallacies, less use of safe gambling practices, and excessive gambling.
Browne and Rockloff [19] ^b^	1524; Australia	This research demonstrated behavioural dependence as unidimensional and distinct from gambling harm. Nonetheless, harm mediated the relationship between behavioural dependence and wellbeing. Taken together, behavioural dependence and the SGHS predicted wellbeing better (10.2% explained variance) than each measure individually.
Dowling et al. [55] ^b^	5000; Tasmania, Australia	The PGSI and SGHS, when considered separately, produced similar low-risk gambling guidelines and captured similar proportions of gamblers in the general population.
Hawker et al. [56] ^b^	97; Tasmania, Australia	The proportion of gamblers who had experienced harm (1+ on the SGHS; 25.77%) was similar to those who had scored 1+ on the PGSI (23.71%).
Hing et al. [57] ^b^	1174; Canada	This study used the SGHS as an outcome measure to develop nine safe gambling practices to best prevent GRH. Six practices were associated with reduced harm (e.g., I keep a household budget) and three were associated with increased harm (e.g., I have used cash advances on my credit card to gamble).
Hing et al. [58] ^b^	92; Victoria, Australia	Gambling harms were negatively associated with saving behaviours related to money management (*r* = −0.34). No significant relationships existed between gambling harm and other aspects of financial literacy/money management (self-confidence, importance, knowledge, helping, and difficulties).
Jenkinson et al. [59] ^b^	5076; Australia	The three most highly endorsed items from the SGHS were reduction of available spending money (24%), reduction of savings (22%), and regrets that made them feel sorry about their gambling (18%).
Newall et al. [60] ^b^	789; UK	Custom sports bettors experienced a higher mean number of gambling harms compared to non-custom sports bettors (2.35 vs. 1.53). The SGHS was also highly correlated with the PGSI (*r^pb^* = 0.82).
Paterson et al. [61] ^b^	5788; Australian Capital Territory, Australia	The 12-month prevalence of experiencing gambling harm was 9.6%. When comparing scores on the SGHS of 1+ (9.6%) to scores of 1+ on the PGSI (10.3%), no statistically significant differences were found. However, 8.7% of non-problem gamblers (PGSI) reported 1+ gambling harms on the SGHS.
Rockloff et al. [62] ^b^	188; US	No significant interactions were found between PGSI status or gambling harm (SGHS) by free-spins influencing bet count.
Rockloff et al. [63] ^b^	7626; Victoria, Australia	The prevalence of experiencing any GRH was 9.6%, with the most frequently endorsed harms being reductions in available spending money (5.1%), reduced savings (3.9%), and regrets about their gambling (3.4%). As the PGSI categories increased, so too did the proportion of having experienced harm. For non-problem gamblers, 4.3% had experienced harm alongside 29.2% of low-risk gamblers, 59.4% of moderate-risk gamblers, and 100% of problem gamblers.
Rodda et al. [64] ^b^	104; Australia	Gamblers who busted (set a limit and broke it) experienced significantly more gambling harms than those who did not bust (4.26 vs. 0.86).
Russell et al. [65] ^b^	2004; New South Wales, Australia	Almost half of the respondents (44.2%) had scored 1+ on the PGSI compared to 45.2% who nominated experiencing some GRH using the SGHS. Furthermore, both PGSI and SGHS scores were significantly associated with exposure to loot boxes.
Russell et al. [66] ^b^	784; Victoria, Australia	Gambling harms were strongly related to the PGSI, with a positive relationship between mean SGHS scores and increasing PGSI categories.
Salonen et al. [67] ^b^	2624; Finland	The prevalence of experiencing GRH was 11%, with emotional/psychological and financial domains of harm being notably impacted.
Woods et al. [68] ^b^	5982; South Australia, Australia	Using the SGHS, the 12-month prevalence of experiencing any gambling harm was 19%, and it was higher among people in Greater Adelaide than the rest of the state.

^a^ evaluations of the SGHS; ^b^ applications of the SGHS.

## Data Availability

Data sharing not applicable to this article.
